# Case Report: Blastic plasmacytoid dendritic cell neoplasm mimicking systemic lymphoma: a multidisciplinary diagnostic challenge

**DOI:** 10.3389/fmed.2026.1934512

**Published:** 2026-09-01

**Authors:** Baixue Lv, Qin Rong, Yan Li, Dongdong Zhang

**Affiliations:** 1 Department of Oncology, Xiangyang No. 1 People’s Hospital, Hubei University of Medicine, Xiangyang, China; 2 Department of Pathology, Xiangyang No. 1 People’s Hospital, Hubei University of Medicine, Xiangyang, China; 3 Department of Pulmonary and Critical Care Medicine, Xiangyang Central Hospital, Hubei University of Arts and Science, Xiangyang, China; 4 Department of Oncology, Xiangyang Central Hospital, Hubei University of Arts and Science, Xiangyang, China

**Keywords:** allo-HSCT, blastic plasmacytoid dendritic cell neoplasm, lymphadenopathy, pruritic erythema, TCF4

## Abstract

**Background:**

Blastic plasmacytoid dendritic cell neoplasm (BPDCN) is an aggressive hematologic malignancy originating from plasmacytoid dendritic cell precursors. While classic cases present with prominent cutaneous nodules and lymphadenopathy, predominant generalized lymphadenopathy can obscure the diagnosis, frequently resulting in a misdiagnosis of systemic lymphoma.

**Case presentation:**

A 56-year-old male presented with a one-month history of generalized pruritic erythema and multi-regional lymphadenopathy. Initial PET-CT demonstrated systemic lymphadenopathy and splenomegaly with increased metabolic activity, mimicking lymphoma. To establish a definitive diagnosis, integrated biopsies of the skin, bone marrow, and inguinal lymph nodes were performed. Bone marrow cytology revealed that unidentified cells accounted for 7% of the total nucleated cells. Immunohistochemical analysis revealed neoplastic cells positive for CD123, CD4, CD56, TCF4, and TCL1, confirming the diagnosis of BPDCN. The patient achieved complete remission (CR) after two cycles of induction chemotherapy with the DA (daunorubicin and cytarabine) regimen, followed by successful consolidation via allogeneic hematopoietic stem cell transplantation (allo-HSCT). At the 4-month post-transplant follow-up, the patient remains in stable remission with no evidence of disease recurrence.

**Conclusion:**

This report details a BPDCN presentation that closely mimics systemic lymphoma. A definitive diagnosis hinges on the integration of multi-site histopathological analysis and comprehensive radiographic findings. Early diagnostic precision is paramount for the timely initiation of intensified therapeutic strategies, such as allo-HSCT, which remain the cornerstone for improving long-term survival in this recalcitrant malignancy.

## Introduction

Blastic plasmacytoid dendritic cell neoplasm (BPDCN) is an exceedingly rare and highly aggressive hematologic malignancy. Historically, the entity was first characterized in 1994 as “agranular CD4-positive natural killer cell leukemia,” a nomenclature reflecting its initially ambiguous immunophenotypic profile (1, 2). Subsequent investigations, however, successfully established its origin from plasmacytoid dendritic cells (pDCs) rather than the natural killer or T-lymphocyte lineages.

The diagnostic framework for BPDCN has been significantly refined in recent years. According to the fifth edition of the World Health Organization (WHO) Classification of Haematolymphoid Tumors (2022), BPDCN is formally categorized under “histiocytic and dendritic cell neoplasms” (3). Diagnosis relies on stringent immunophenotypic criteria: beyond the expression of CD4 and/or CD56, it necessitates positivity for CD123 along with at least one additional pDC-associated marker (e.g., TCF4, TCL1, CD303, or CD304), or the simultaneous expression of any three of these four pDC-specific markers. Furthermore, the definitive exclusion of lineage-specific markers—including myeloid-associated (CD14, CD34, lysozyme, and MPO) and lymphoid-associated (CD3 and CD19) antigens—is mandatory to establish a precise diagnosis (4).

Epidemiologically, BPDCN exhibits an exceptionally low incidence of approximately 0.04 per 100,000 individuals, demonstrating a distinctive bimodal age distribution with peaks in adolescents (<20 years) and older adults (>60 years). With a marked male predominance (approximately 2:1), the disease typically involves the bone marrow, lymph nodes, and skin (5). Cutaneous involvement is classically regarded as the hallmark initial manifestation, often presenting as solitary or multiple nodules and violaceous plaques localized to sun-exposed areas such as the scalp, face, and upper chest (6). However, the clinical heterogeneity of BPDCN often poses significant diagnostic challenges. This report details a case of BPDCN in a 56-year-old man presenting primarily with generalized lymphadenopathy and only subtle cutaneous lesions. This clinical presentation stands in stark contrast to the characteristic skin-predominant onset typically associated with this entity, highlighting a critical diagnostic pitfall and underscoring the necessity of an integrated diagnostic approach involving radiographic imaging and multi-site histopathological analysis.

## Case report

**Figure 1:**
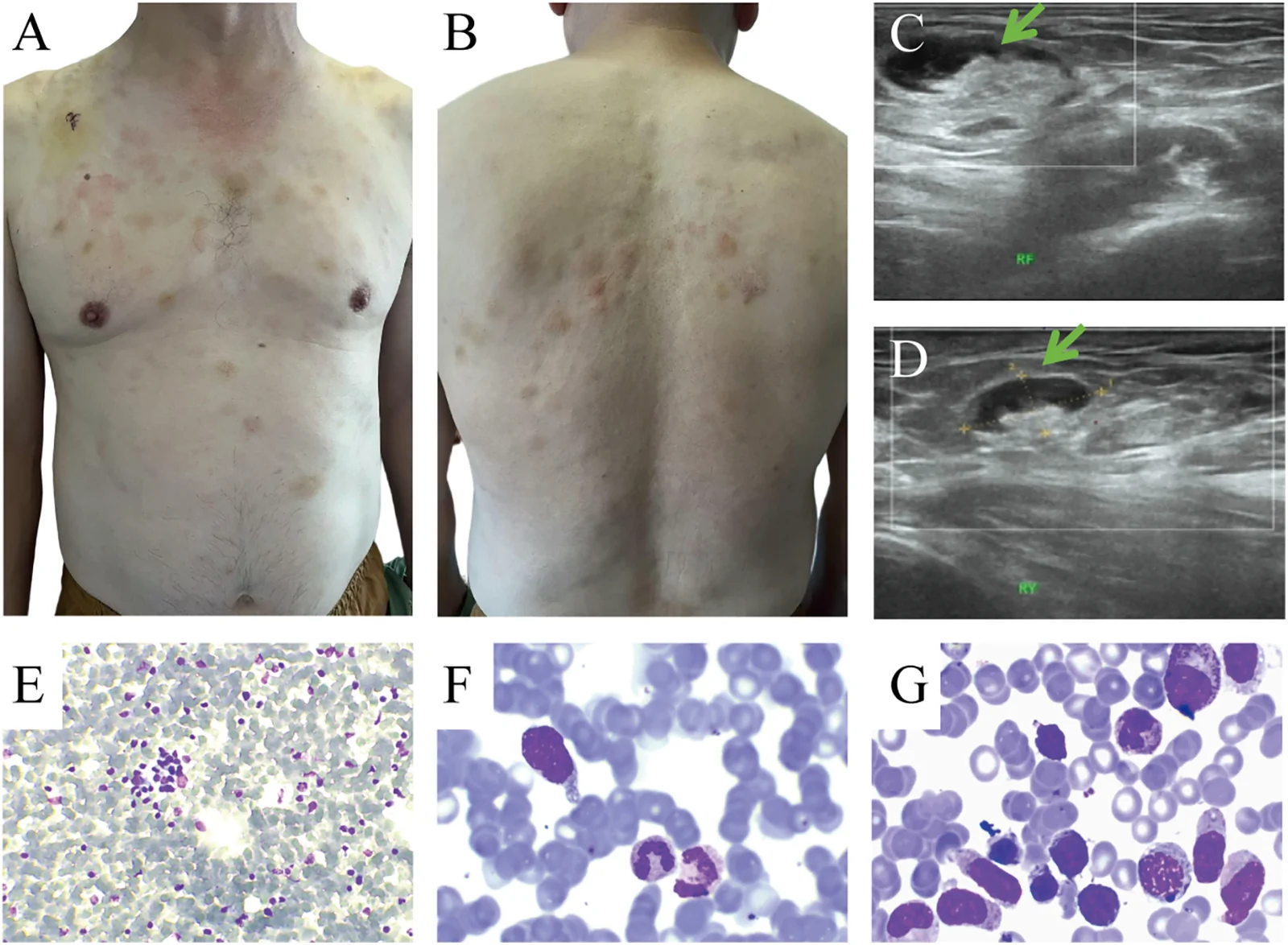
Clinical manifestations, ultrasonography, and cytomorphological findings. **(A,B)** Widespread, ecchymosis-like erythematous plaques involving the anterior chest and back. **(C,D)** Ultrasonographic images of the right inguinal and axillary regions revealing significant lymphadenopathy with concentric cortical thickening (indicated by arrows). **(E)** Fine-needle aspiration (FNA) cytology of the right upper arm mass showing scattered neoplastic lymphoid cells against a background of erythrocytes. **(F,G)** Bone marrow aspirate smear (Wright-Giemsa stain) showing atypical mononuclear cells (approximately 7%) characterized by scant basophilic cytoplasm and irregular nuclear contours.

A 56-year-old male was admitted to our institution with a one-month history of generalized pruritic erythema and multi-regional lymphadenopathy. Relevant medical history included type 2 diabetes mellitus treated with subcutaneous dulaglutide, coronary heart disease receiving aspirin and rosuvastatin, and a total thyroidectomy. He reported no hypertension, drug allergies, or constitutional symptoms such as fever, night sweats, or weight loss. His baseline Karnofsky Performance Status (KPS) was 100. Physical examination upon admission revealed multiple palpable, enlarged lymph nodes in the cervical, supraclavicular, and inguinal regions **(**Figures 1A,B**)**, accompanied by generalized ecchymoses and erythema involving the anterior chest and back. Furthermore, several subcutaneous nodules of varying sizes were palpable on the right upper arm; these nodules were firm, non-tender, and lacked localized erythema or swelling. Laboratory investigations were largely unremarkable, except for a marginal elevation in total bile acids. Initial ultrasonography identified systemic lymphadenopathy with localized cortical thickening in the bilateral inguinal **(**Figures 1C,D**)**, cervical, and axillary regions. An ultrasound-guided fine-needle aspiration (FNA) was performed on a subcutaneous mass of the right upper arm, cytological analysis revealed scattered lymphoma-like neoplastic cells against a background of erythrocytes **(**Figure 1E**).** Bone marrow aspirate cytology revealed unclassifiable cells comprising 7% of the total nucleated population (Figures 1F,G**)**. However, owing to institutional limitations regarding bone marrow flow cytometry at the time of diagnostic workup, specific immunophenotypic characterization of these cells was not performed. Consequently, it remains undetermined whether this fraction represented true neoplastic bone marrow involvement or secondary reactive precursors.

**Figure 2:**
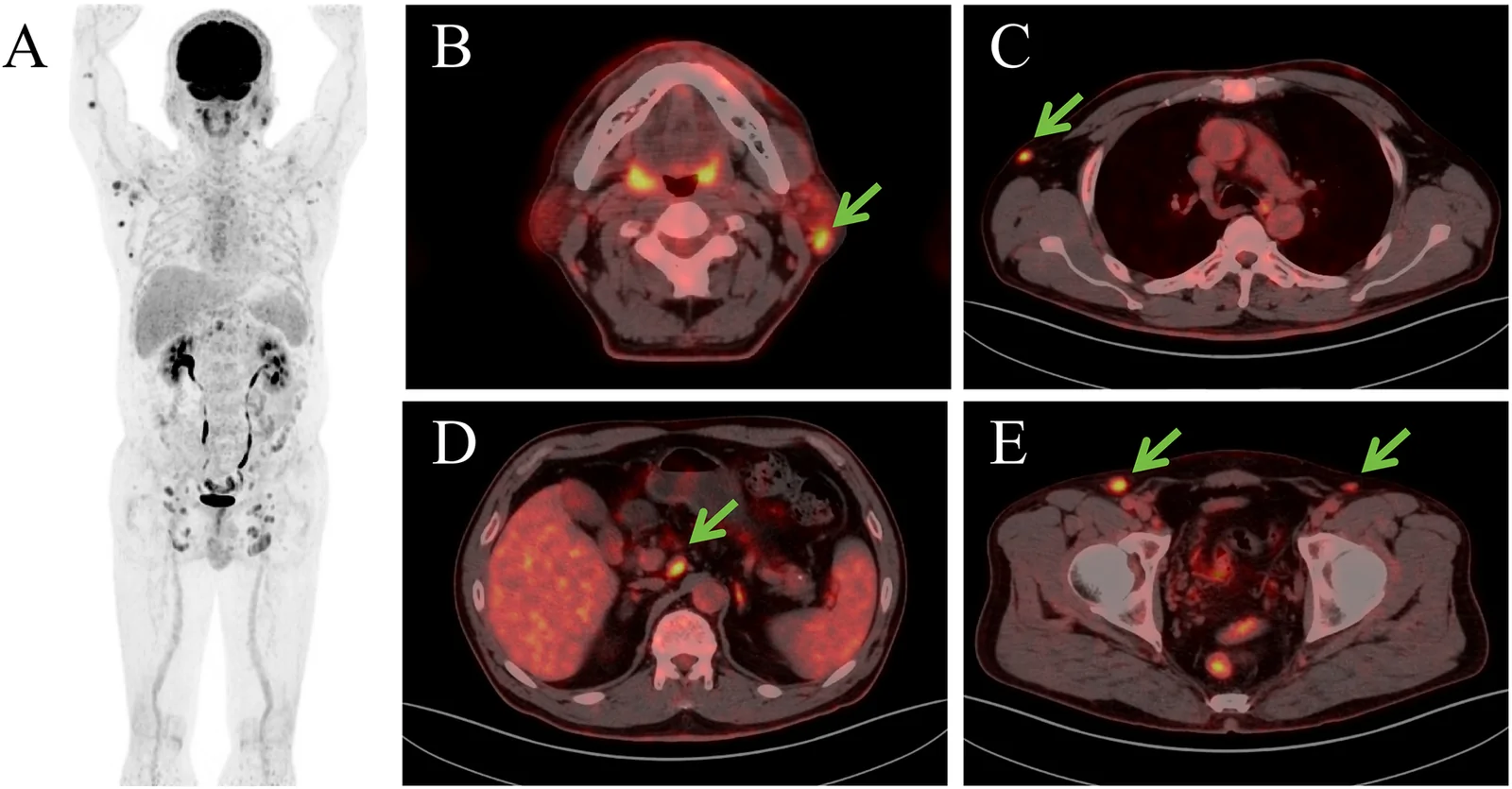
Whole-body ^18^F-FDG PET/CT imaging. **(A)** Maximum intensity projection (MIP) image demonstrating multisite hypermetabolic foci localized to the cervical, axillary, retroperitoneal, and inguinal regions. **(B–E)** Representative axial fused PET/CT images identifying intensely FDG-avid lymph nodes (arrows) in the left postauricular region **(B)**, right axilla **(C)**, para-aortic/retroperitoneal space **(D)**, and bilaterally in the inguinal regions **(E).**

**Figure 3:**
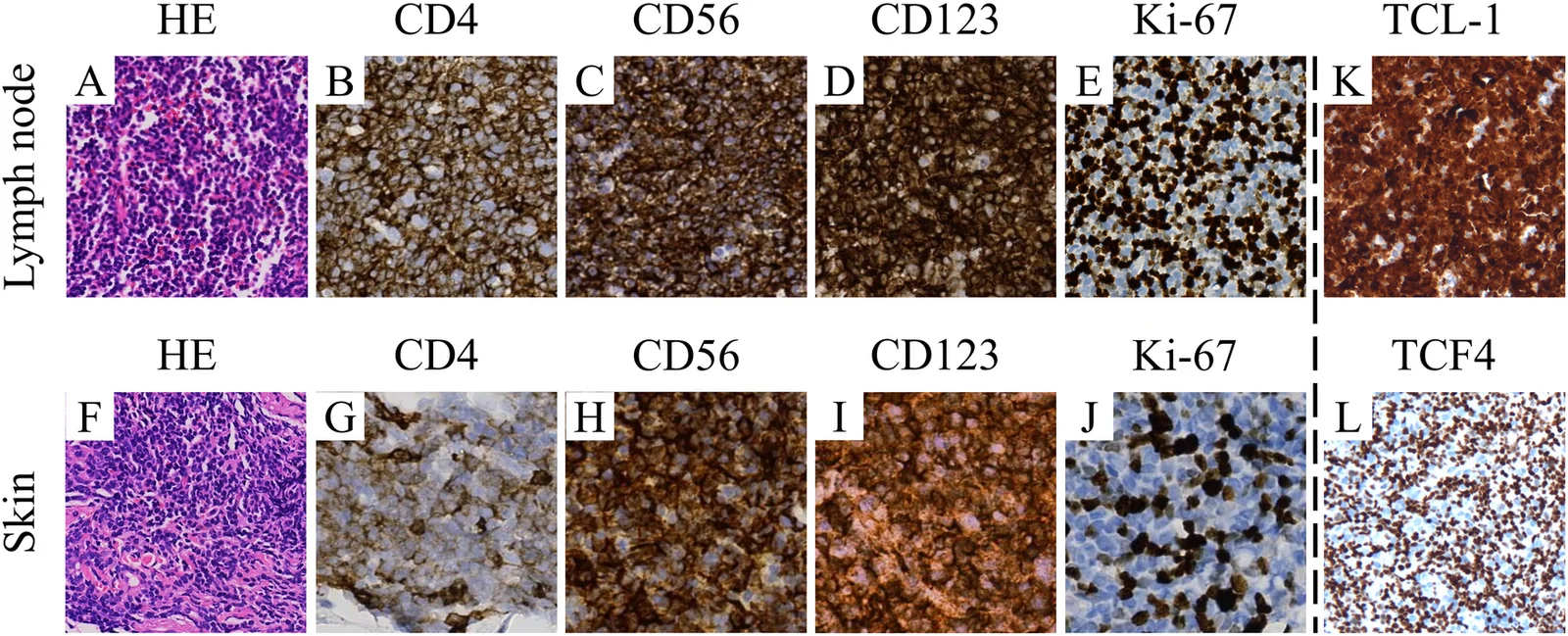
Histopathological and immunohistochemical (IHC) characterization of inguinal lymph node and chest skin biopsies. **Upper panel (A–E):** Inguinal lymph node; **Lower panel (F–J):** Anterior chest skin. **(A–F)** Hematoxylin and eosin (H&E) staining (100×) revealing diffuse, non-cohesive infiltration of medium-sized neoplastic lymphoid cells. **(B–D and G–I)** IHC profiling (200×) demonstrating strong and diffuse cytoplasmic/membranous expression of CD4, CD56, and CD123 in both lymph node and cutaneous tissues. **(E–J)** Ki-67 proliferation index, estimated at approximately 40% in the lymph node **(E)** and 70% in the skin lesion **(J)**. **(K,L)** Confirmatory IHC staining (200×) of the lymph node showing nuclear positivity for TCL-1 **(K)** and TCF4 **(L)**, supporting the diagnosis of BPDCN.

Subsequent ^18^F-fluorodeoxyglucose (FDG) positron emission tomography-computed tomography (PET-CT) demonstrated generalized hypermetabolic lymphadenopathy, splenomegaly, and increased metabolic activity within multiple subcutaneous foci and the bone marrow cavity (Figures 2A–E). These systemic findings were initially highly suggestive of advanced-stage lymphoma. Definitive diagnosis was established through comprehensive morphological assessment and immunohistochemical (IHC) profiling (Figure 3). The neoplastic cells exhibited robust and diffuse positivity for pathognomonic BPDCN markers, specifically CD4, CD56, CD123, TCF4, and TCL1. Furthermore, the Ki-67 proliferation index was significantly elevated in both the cutaneous and lymph node specimens. To ensure diagnostic precision and exclude mimetic entities, an extensive panel of lineage-specific markers was utilized: T-cell markers (CD3, CD2, CD5, CD7, CD8), B-cell markers (CD20, PAX-5), myeloid markers (CD34, CD117), and plasma cell markers (CD138, MUM-1) were all negative (Supplementary Figure S1). By integrating the radiographic suspicion with these distinctive immunophenotypic features, a definitive diagnosis of BPDCN was established. However, it should be noted that no cytogenetic or molecular genomic studies were performed, which precludes a deeper molecular characterization of this specific case.

**Figure 4:**
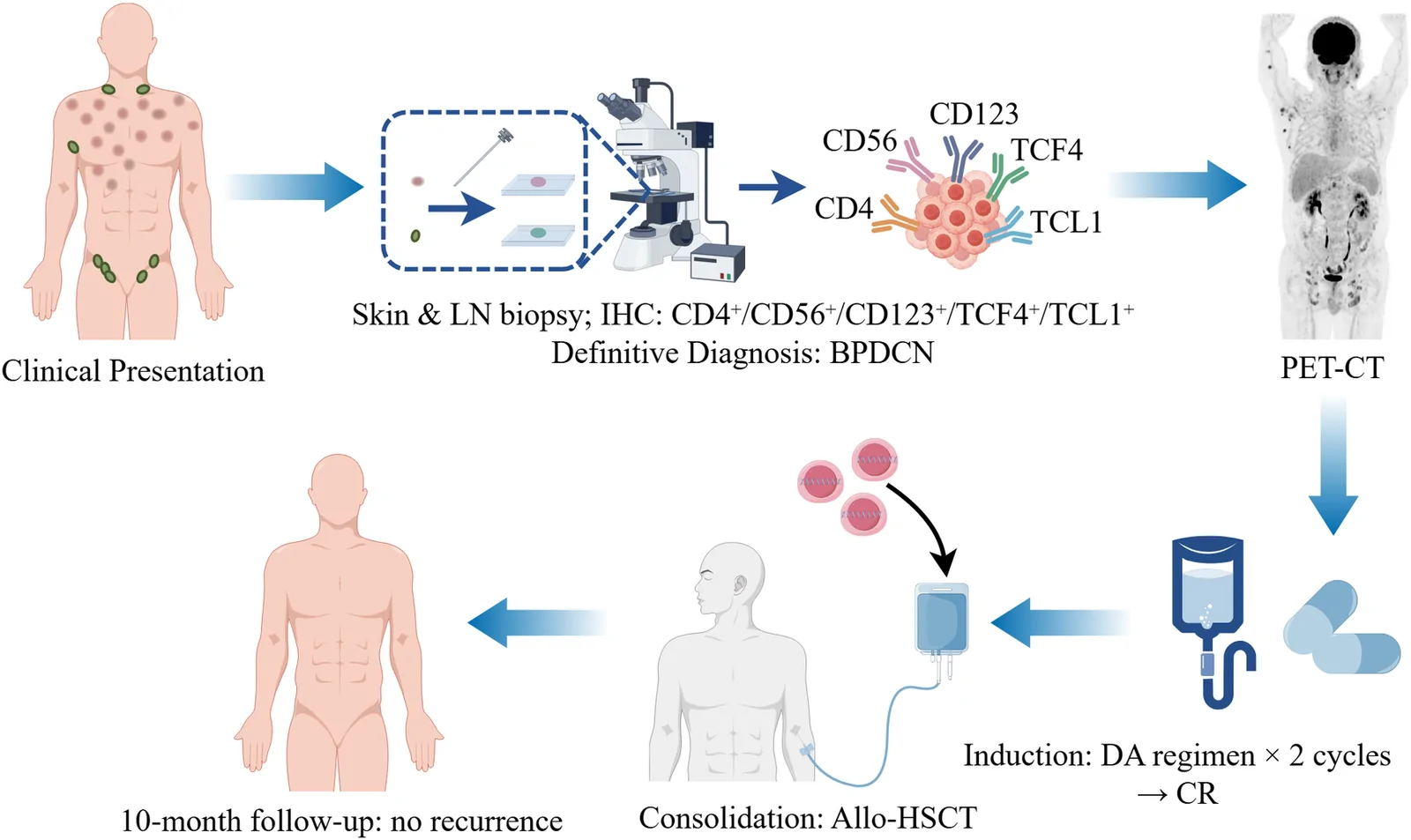
Clinical diagnostic and therapeutic trajectory. BPDCN, blastic plasmacytoid dendritic cell neoplasm; DA, daunorubicin and cytarabine; CR, complete remission; Allo-HSCT, allogeneic hematopoietic stem cell transplantation.

Following diagnosis, the patient was transferred to another facility, where he received two cycles of DA (daunorubicin plus cytarabine) induction as a bridge to allogeneic hematopoietic stem cell transplantation (allo-HSCT). As ascertained via telephone follow-up, he experienced marked regression of lymphadenopathy, and a subsequent post-treatment bone marrow assessment revealed the complete clearance of the previously noted unclassifiable cells. To date, over a 10-month follow-up period, he remains healthy with no evidence of disease relapse. The detailed diagnostic and treatment algorithm is presented in Figure 4**.**

## Discussion

In this report, we detail the diagnostic workup and clinical management of a BPDCN case characterized by prominent lymphadenopathy and minimal cutaneous involvement. While cutaneous lesions—typically violaceous nodules or plaques—are the hallmark initial manifestation in 77% to 95% of patients, approximately 10% to 25% of cases present with clinically occult skin involvement at early stages Our patient's presentation, dominated by systemic lymphadenopathy and splenomegaly with only subtle ecchymoses, represents a significant “diagnostic decoy.” This highlights the necessity of maintaining a high index of suspicion for BPDCN even in the absence of classic dermatological findings to avoid misdiagnosis as systemic lymphoma or acute leukemia.

BPDCN arises from the precursors of plasmacytoid dendritic cells (pDCs), which normally migrate to skin tissues only upon inflammatory challenge. The interindividual heterogeneity in tissue infiltration suggests that BPDCN cells may either retain specific migratory patterns of normal pDCs or reflect varying stages of disease progression (7). The diagnostic framework for BPDCN has been significantly refined in the 2022 WHO classification. CD123 (interleukin-3 receptor *α*-chain) is the pathognomonic surface marker for the pDC lineage and serves as both a diagnostic anchor and a therapeutic target. Specifically, TCF4 is a master regulator of pDC development, providing superior diagnostic specificity over more traditional markers (3). In this case, initial immunohistochemistry was uniformly negative for lineage-specific and cytotoxic markers, effectively excluding B-cell, T-cell, NK-cell, and myeloid neoplasms. Conversely, the neoplastic cells co-expressed CD123, CD4, and CD56, indicating a plasmacytoid dendritic cell derivation. This immunophenotype prompted confirmatory staining for the highly specific markers TCF4 and TCL1, both of which were positive. These concordant findings across two independent biopsy sites (inguinal lymph node and skin), alongside negative EBER *in situ* hybridization to exclude EBV-driven lymphoproliferative disorders, definitively establish the diagnosis of BPDCN according to current WHO criteria (3).

While the systemic onset of BPDCN is well-documented (8), our case perfectly illustrates the profound diagnostic pitfalls this deceptive entity presents in clinical practice. The patient exhibited atypical bruise-like patches rather than the hallmark violaceous nodules (9), and the widespread FDG-avid lymphadenopathy with splenomegaly closely masqueraded as advanced-stage lymphoma. The primary message of this case is to emphasize that such an elusive clinical phenotype exists and can easily lead to misdiagnosis. Therefore, it is imperative for clinicians to maintain a high index of suspicion, and it remains the critical prerogative of histopathologists to correctly identify this malignancy through comprehensive immunophenotypic profiling.

Optimal frontline therapy for BPDCN has evolved substantially over the past decade. Historically, lymphoma-like regimens (e.g., CHOP) were commonly employed but were associated with transient responses and a median overall survival of only 12 to 14 months (4). Current clinical evidence suggests that intensive leukemia-like induction regimens yield superior complete response (CR) rates. While ALL-like protocols (e.g., Hyper-CVAD) often achieve high initial CR, they are associated with significant treatment-related toxicity. Conversely, AML-like regimens (e.g., the DA regimen used in this case) provide a viable alternative for achieving rapid systemic clearance and bone marrow conversion (10). A pivotal advancement in the BPDCN landscape is Tagraxofusp (SL-401), a CD123-targeted recombinant fusion protein that has demonstrated an overall response rate of 90% in treatment-naïve patients, acting as an effective bridge to definitive consolidation (11, 12).

Despite high initial response rates, BPDCN is characterized by an exceptionally high frequency of relapse. Allogeneic HSCT currently represents the only therapeutic modality with curative potential. Data consistently show that patients undergoing allo-HSCT in their first CR (CR1) achieve a 3-year overall survival rate exceeding 60%, which is markedly superior to chemotherapy alone (13, 14). Furthermore, emerging combination therapies, such as venetoclax with hypomethylating agents, offer promising results for elderly or medically unfit patients (15, 16). Furthermore, emerging therapeutic modalities-including CD123-targeted chimeric antigen receptor T-cell (CAR-T) therapy, BET inhibitors, and PD-1 inhibitors-are currently under active investigation (17, 18).

While the patient achieved a favorable initial response, a primary limitation of this report is the absence of granular treatment data. Because the patient was transferred to an outside facility immediately following diagnosis, detailed therapeutic parameters and standardized post-treatment evaluations were unavailable. Consequently, clinical outcomes were ascertained solely via telephone follow-up. First, as a single-case report, the findings and therapeutic outcomes lack broader generalizability; the inherent heterogeneity of BPDCN means that these observations may not be representative of the entire patient population. Second, the optimal duration and intensity of induction chemotherapy prior to consolidation remain a subject of clinical debate. Following a prompt clinical response to just two cycles of DA induction, the patient was successfully bridged to consolidative allo-HSCT. Although early transplantation in the first CR (CR1) is widely regarded as a critical prognostic factor, it remains unclear whether a more prolonged induction phase would have further consolidated the molecular remission or conversely increased treatment-related toxicity and delayed the window for curative transplant. Third, the selection of consolidation therapy was influenced by regional drug accessibility. While CD123-targeted agents, such as tagraxofusp, have demonstrated remarkable efficacy and are increasingly incorporated into frontline management in Western countries, these agents are currently unavailable in China. Consequently, allo-HSCT remains the primary and most accessible curative option in our clinical setting. Further studies are needed to compare the long-term outcomes of targeted maintenance versus traditional cellular therapy in such populations. An additional constraint is the absence of molecular and cytogenetic profiling, such as karyotyping and next-generation sequencing, precluding a deeper genomic characterization of this case. Lastly, while the patient has maintained clinical stability for 10 months post-transplantation, this follow-up period remains relatively brief. Given the notoriously high risk of late relapse in BPDCN, extended longitudinal surveillance is critical to confirm the durability of remission and to monitor for late-onset allo-HSCT complications.

In conclusion, this case exemplifies an atypical clinical presentation of BPDCN defined by extensive lymphadenopathy and subtle skin involvement. The definitive diagnosis was established through the integration of PET-CT imaging and comprehensive immunophenotypic profiling of the CD123/TCF4/TCL1 axis across multiple biopsy sites. Clinical success was achieved using a DA-based induction regimen as a bridge to curative allo-HSCT. The standardized diagnostic-therapeutic algorithms are essential strategies for enhancing the long-term survival and prognosis of patients with this highly malignant disease.

## Data Availability

The original contributions presented in the study are included in the article/Supplementary Material, further inquiries can be directed to the corresponding authors.

## Supplementary material

Supplementary Figure S1 Supplementary immunohistochemical analysis (200×)
